# Performance of lung ultrasound in the diagnosis of pediatric pneumonia in Mozambique and Pakistan

**DOI:** 10.1002/ppul.25176

**Published:** 2020-11-26

**Authors:** Amy Sarah Ginsburg, Jennifer L. Lenahan, Fyezah Jehan, Rubao Bila, Alessandro Lamorte, Jun Hwang, Lola Madrid, Muhammad Imran Nisar, Pio Vitorino, Neel Kanth, Reyes Balcells, Benazir Baloch, Susanne May, Marta Valente, Rosauro Varo, Naila Nadeem, Quique Bassat, Giovanni Volpicelli

**Affiliations:** ^1^ Clinical Trial Center University of Washington Seattle Washington USA; ^2^ Save the Children Federation, Inc. Seattle Washington USA; ^3^ Department of Pediatrics and Child Health Aga Khan University Karachi Pakistan; ^4^ Centro de Investigação em Saúde de Manhiça (CISM) Maputo Mozambique; ^5^ Department of Emergency Medicine Parini Hospital Aosta Italy; ^6^ ISGlobal, Hospital Clínic, Universitat de Barcelona Barcelona Spain; ^7^ Sindh Government Children's Hospital–Poverty Eradication Initiative Karachi Pakistan; ^8^ Department of Radiology Aga Khan University Karachi Pakistan; ^9^ Institució Catalana de Recerca i Estudis Avançats (ICREA) Barcelona Spain; ^10^ Department of Pediatrics, Hospital Sant Joan de Deu Universitat de Barcelona Barcelona Spain; ^11^ Consorcio de Investigacion Biomedica en Red de Epidemiologia y Salud Publica (CIBERESP) Madrid Spain; ^12^ Department of Emergency Medicine San Luigi Gonzaga University Hospital Orbassano Italy

**Keywords:** chest ultrasound, childhood pneumonia, interrater reliability, low‐resource settings

## Abstract

**Introduction:**

Improved pneumonia diagnostics are needed in low‐resource settings (LRS); lung ultrasound (LUS) is a promising diagnostic technology for pneumonia. The objective was to compare LUS versus chest radiograph (CXR), and among LUS interpreters, to compare expert versus limited training with respect to interrater reliability.

**Methods:**

We conducted a prospective, observational study among children with World Health Organization (WHO) Integrated Management of Childhood Illness (IMCI) chest‐indrawing pneumonia at two district hospitals in Mozambique and Pakistan, and assessed LUS and CXR examinations. The primary endpoint was interrater reliability between LUS and CXR interpreters for pneumonia diagnosis among children with WHO IMCI chest‐indrawing pneumonia.

**Results:**

Interrater reliability was excellent for expert LUS interpreters, but poor to moderate for expert CXR interpreters and onsite LUS interpreters with limited training.

**Conclusions:**

Among children with WHO IMCI chest‐indrawing pneumonia, expert interpreters may achieve substantially higher interrater reliability for LUS compared to CXR, and LUS showed potential as a preferred reference standard. For point‐of‐care LUS to be successfully implemented for the diagnosis and management of pneumonia in LRS, the clinical environment and amount of appropriate user training will need to be understood and addressed.

## 1 INTRODUCTION

Each year, approximately 920,000 children die before their fifth birthdays due to pneumonia.^1^ Greater access to appropriate and effective pneumonia diagnostics, particularly in low‐resource settings (LRS), is critical to addressing child mortality. In LRS, pneumonia is identified using the World Health Organization (WHO) Integrated Management of Childhood Illness (IMCI) guidelines that depend on assessing variable and subjective clinical signs, specifically respiratory rate and chest indrawing.^2^ It is not clear how effective WHO IMCI guidelines are in identifying pneumonia,^3^ and because the guidelines prioritize diagnostic sensitivity over specificity, there is concern regarding antimicrobial overuse and resistance.^4^ Diagnostic alternatives to WHO IMCI also have challenges.^5^ Clinical diagnosis not using WHO IMCI guidelines lack standardization. If available, chest radiographs (CXR) can be expensive, difficult to obtain, time‐consuming, and expose the child to ionizing radiation.^5^, ^6^, ^7^ Microbiology (e.g., blood, lung/pleural aspiration, and/or bronchoalveolar lavage culture) is invasive, slow, and detects a limited proportion of cases.^5^ Biomarkers such as C‐reactive protein can correlate with bacterial infection but do not have a set threshold nor indicate a specific etiology.^5^ Given these limitations and that diagnostic tests used for pediatric pneumonia have not been sufficiently validated despite their routine use, there is no satisfying safe and effective reference standard for the accurate and reliable diagnosis of pediatric pneumonia.^8^ Lung ultrasound (LUS) is a promising technology that can dynamically visualize the lungs with potentially high diagnostic accuracy for pneumonia.^6^ Advantages of LUS, relative to CXR, include its lower cost, portability, ease of use, and absence of ionizing radiation.^6^, ^7^, ^9^ We conducted a pilot study in Mozambique and Pakistan to investigate the use of point‐of‐care LUS as a tool for the diagnosis of pediatric pneumonia in LRS among children with WHO IMCI chest‐indrawing pneumonia.

## 2 METHODS

### 2.1 Study design, setting, and participants

The methods of this study have been described previously.^10^ The primary aim of this prospective facility‐based cohort study is to provide evidence regarding the use of LUS as a diagnostic tool for pneumonia in children presenting to district hospitals in Manhiça, Mozambique and Karachi, Pakistan. We investigated whether interrater reliability was similar among LUS interpreters and among CXR interpreters.

**Figure 1:**
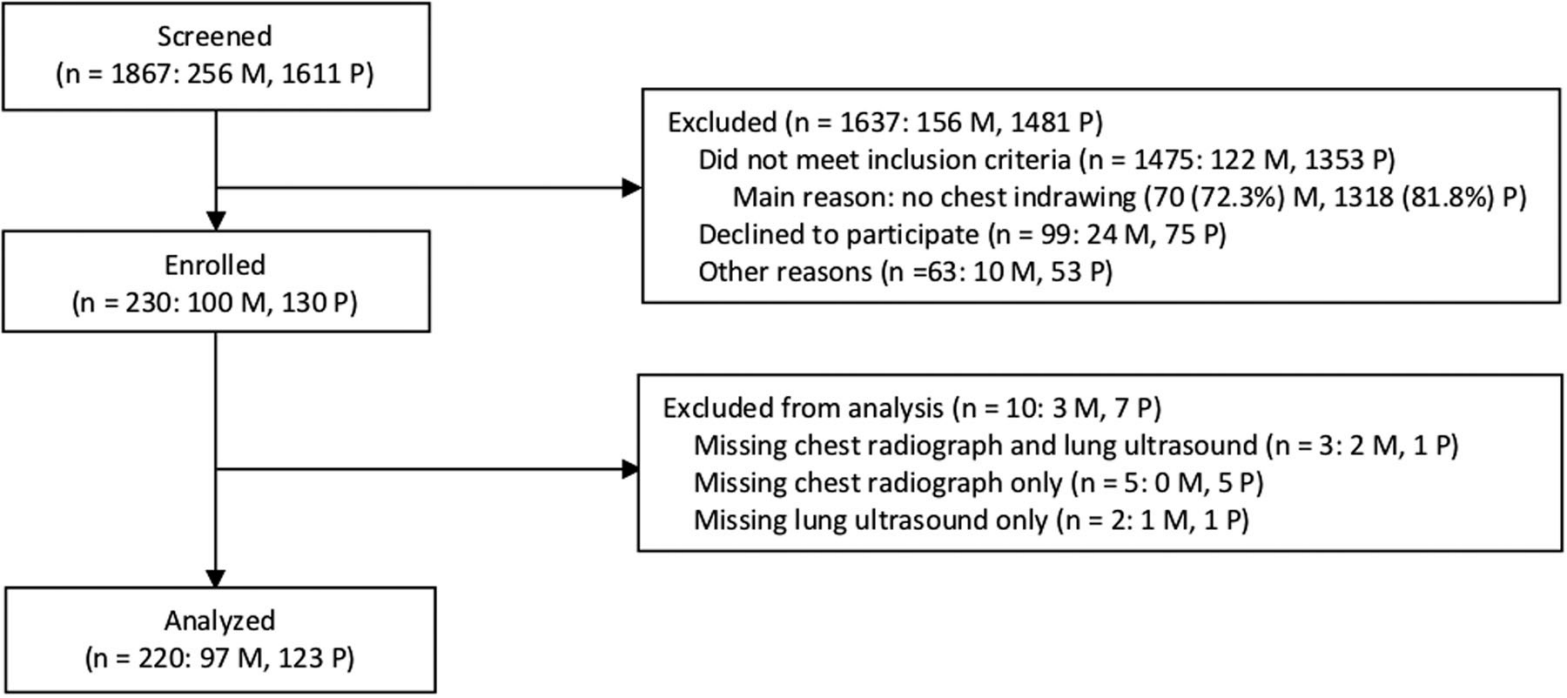
Flowchart of study participants by country: Mozambique (M), Pakistan (P)

Children aged 2–23 months meeting the WHO IMCI chest‐indrawing pneumonia case definition in the outpatient and/or emergency departments of Manhiça District Hospital, a low‐volume, rural hospital in Manhiça and Sindh Government Children's Hospital–Poverty Eradication Initiative, a high‐volume, urban hospital in Karachi, were screened by study staff to determine eligibility (Table 1; Figure 1). The study was conducted in accordance with the International Conference on Harmonisation, Good Clinical Practice, and the Declaration of Helsinki 2008, and was approved by the Western Institutional Review Board in the state of Washington; the Comité Institucional de Bioética em Saúde do Centro de Investigação em Saúde de Manhiça (Manhiça); the Comité Nacional de Bioética em Saúde (Maputo, Mozambique; Ref. 246/CNBS/17); the Comite de Ética del Hospital Clínic de Barcelona (Barcelona, Spain); and the Aga Khan University Ethics Review Committee (Karachi). This study was registered NCT03187067 with ClinicalTrials.gov.

**Table 1:** Study definitions and eligibility criteria

**Definitions**	
Fast breathing for age	Children 2 to <12 months of age: RR ≥50 breaths per minute Children ≥12 months of age: RR ≥40 breaths per minute
Severe respiratory distress	Grunting, nasal flaring, and/or head nodding
WHO IMCI general danger signs	Lethargy or unconsciousness, convulsions, vomiting everything, inability to drink or breastfeed
Eligibility criteria	
Inclusion criteria	2–23 months of age Cough <14 days or difficulty breathing Visible indrawing of the chest wall with or without fast breathing for age Ability and willingness of child's caregiver to provide informed consent and to be available for follow‐up for the planned duration of the study, including accepting a home visit if he/she fails to return for a scheduled study follow‐up visit
Exclusion criteria	Resolution of chest indrawing after bronchodilator challenge, if wheezing at screening examination Severe respiratory distress arterial Spo_2 _<90% in room air, as assessed noninvasively by a pulse oximeter WHO IMCI general danger signs Stridor when calm Known or possible tuberculosis (history of a cough ≥14 days) Any medical or psychosocial condition or circumstance that, in the opinion of the investigators, would interfere with the conduct of the study or for which study participation might jeopardize the child's health Living outside the study catchment area

Abbreviations: IMCI, Integrated Management of Childhood Illness; RR, respiratory rate; SpO_2_, oxyhemoglobin saturation; WHO, World Health Organization.

### 2.2 Study procedures

On Day 1, after enrollment, eligible children underwent a history and physical examination as well as CXR and LUS collection. All enrolled children received a local standard of care without the results of the LUS examinations informing clinical care.

**Figure 2:**
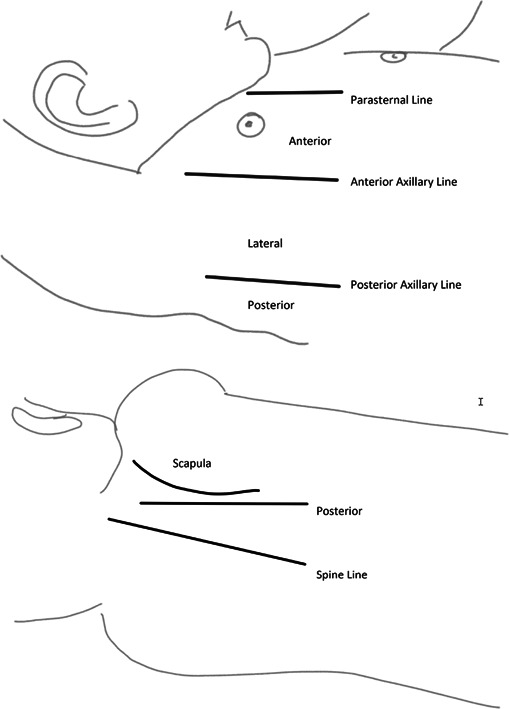
Lung ultrasound examinations consisted of longitudinal and oblique scans obtained of the anterior, lateral, and posterior sides of the child's chest

LUS examinations (longitudinal and oblique scans obtained of the anterior, lateral, and posterior sides of the child's chest [Figure 2]) were performed by nonphysician healthcare personnel (a nurse and a medical agent in Mozambique, and two radiology technicians in Pakistan) who received a 1‐day standardized training course as well as 3 days of supervised practice before the initiation of study activities. LUS interpretation using a standardized scoresheet targeted the detection of typical lung consolidations and/or pleural effusions. At least two independent physicians extensively trained in LUS (expert LUS interpreters) and blinded to clinical presentation interpreted each examination. If discordant, a designated expert LUS interpreter acted as a tiebreaker. LUS operators at each site also independently from one another interpreted LUS scans in batches at a later time using the same standardized scoresheet.

CXR images were collected based on the standard practice at each study site. A CXR interpretation panel of six expert interpreters, comprised of four radiologists, one pediatric pulmonologist, and one pediatric infectious diseases physician, investigated radiographic indicators of primary endpoint pneumonia, in a process modeled after the WHO CXR standardized interpretation process which focused on the presence of consolidation, infiltrates, and/or effusion.^11^, ^12^, ^13^, ^14^ To qualify as an expert CXR interpreter, each member of the panel had trained in and previously performed WHO CXR interpretation, and in preparation for this study, achieved a score of at least 80% sensitivity and 80% specificity in the interpretation of a testing set of 25 CXRs from the WHO CXR in epidemiological studies series. For a final expert CXR diagnosis, at least three members of the study's CXR interpretation panel independently interpreted each CXR. In situations where there were more than three interpreters, three interpretations were randomly selected, and if the first two interpretations were discordant, the third would act as a tiebreaker.

### 2.3 Outcomes

The primary outcome was LUS findings among children with WHO IMCI chest‐indrawing pneumonia upon enrollment. We focus here on pneumonia as assessed by expert LUS interpreters, LUS interpreters with limited training, and expert CXR interpreters, and compare interrater reliability between these interpreters.

### 2.4 Statistical analysis

Agreement of the LUS and CXR imaging modalities regarding the primary endpoint of pneumonia was estimated using Cohen's *κ*, based on the expert LUS interpreters, LUS interpreters with limited training, and expert CXR interpreters. For both LUS and CXR images, expert interpreters were compared to each other. For LUS images, expert interpreters were also compared to onsite interpreters with limited training. All analyses were performed using R (version 3.5.1; R Foundation for Statistical Computing).

## 3 RESULTS

**Figure 3:**
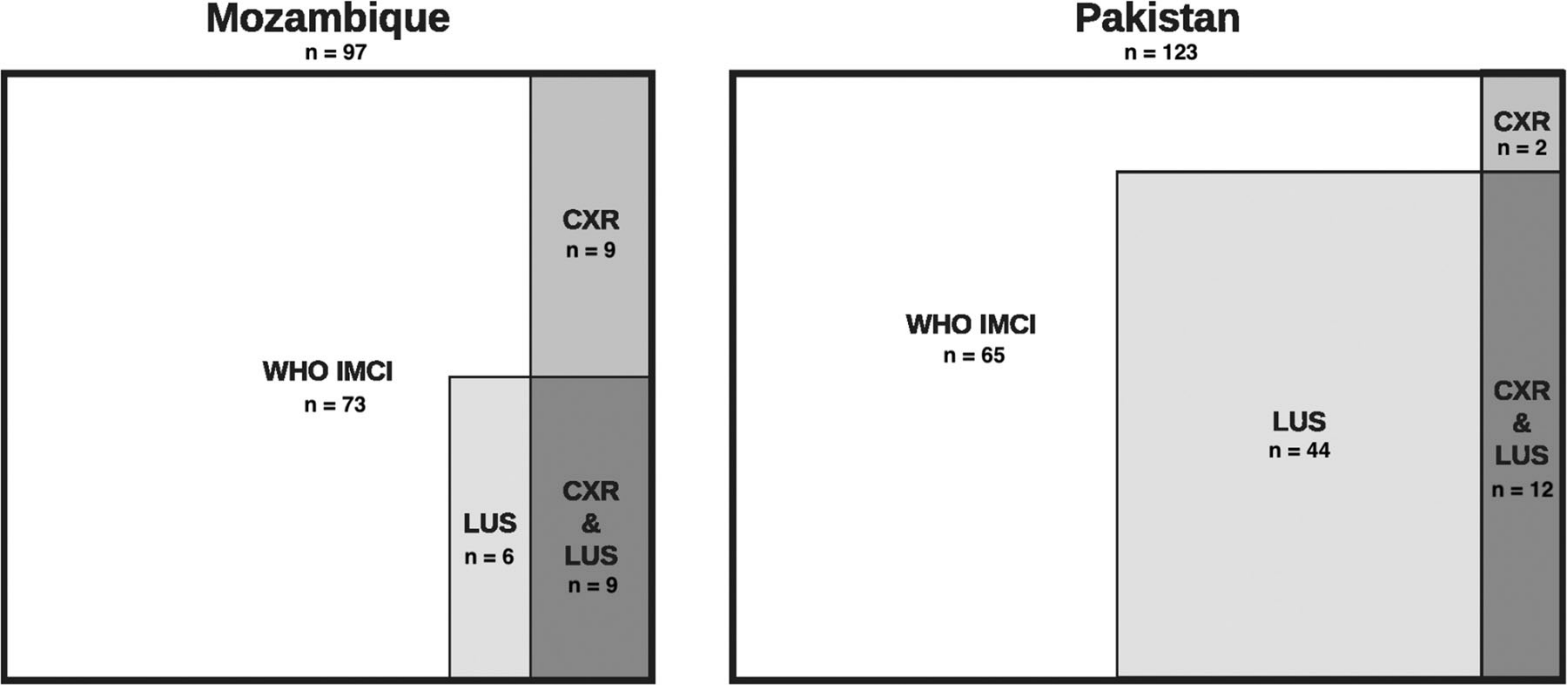
Graphical representation of a number of children diagnosed with chest‐indrawing pneumonia by World Health Organization (WHO) Integrated Management of Childhood Illness (IMCI) criteria, chest radiograph (CXR), and/or lung ultrasound (LUS) in Mozambique and Pakistan

Enrollment began in August 2017 in Mozambique and October 2017 in Pakistan. The last visits were completed in June 2018 in Mozambique and April 2018 in Pakistan. In total, 1867 (256 in Mozambique; 1611 in Pakistan) children were screened, of which 230 were enrolled, 1475 were ineligible, 99 were eligible but refused enrollment consent, and 63 were not enrolled for other reasons (e.g., the caregiver was under 18 years of age; Figure 1). The most frequent reason for ineligibility at both sites was a lack of chest indrawing (70 in Mozambique; 1318 in Pakistan). LUS and CXR imaging were available for 220 children. Baseline characteristics of children with WHO IMCI chest‐indrawing pneumonia are presented by country in Table 2. Numbers of LUS and CXR pneumonia determinations as classified by expert LUS and expert CXR interpreters are presented by country in Table A1 and graphically in Figure 3. LUS identified 9 of 18 (Mozambique) and 11 of 13 (Pakistan) CXR‐confirmed cases and identified 6 (Mozambique) and 45 (Pakistan) additional cases not confirmed by CXR. CXR‐confirmed pneumonia was identified in 18.6% (18/97) of children in Mozambique and in 10.6% (13/123) of children in Pakistan. The agreement between LUS and CXR was poor to moderate (*κ* = 0.178 for Pakistan and *κ* = 0.453 for Mozambique).

**Table 2:** Baseline characteristics of children with World Health Organization Integrated Management of Childhood Illness chest‐indrawing pneumonia at enrollment by country

	Mozambique	Pakistan
	*N* = 97	*N* = 123
Age (months)		
Mean (*SD*)	10.90 (6.02)	6.65 (4.68)
<12, *n* (%)	54 (55.7)	108 (87.8)
Female, *n* (%)	39 (40.2)	31 (25.2)
Temperature (°C), mean (*SD*)	37.06 (1.09)	36.73 (0.76)
Fever (≥38°C), *n* (%)	21 (21.6)	11 (8.9)
Respiratory rate (breaths/min)		
<12 months, mean (*SD*)	52.87 (11.20)	53.44 (7.92)
≥12 months, mean (*SD*)	44.26 (10.00)	48.13 (10.06)
Tachypnea, *n* (%)	52 (53.6)	79 (64.2)

As shown in Table 3a, the expert LUS interpreters demonstrated excellent interrater reliability with *κ* = 0.914, while the interrater reliability among onsite and between onsite and expert LUS interpreters varied substantially (*κ* from 0.196 to 0.983; cross‐classified counts comparing LUS interpreters are shown in Tables A2, A3, A4). Onsite LUS interpreters in Mozambique diagnosed pneumonia 0.5 times or less frequently than expert LUS interpreters (7.2% and 2.1% for onsite interpreters A and B vs. 15.5% for final expert LUS interpretation). Onsite LUS interpreters in Pakistan diagnosed pneumonia about 1.4 times more frequently than expert LUS interpreters (62.6% and 63.4% for onsite interpreters C and D vs. 45.5% for final expert LUS interpretation; Table A3). As shown in Table 3b, the interrater reliability observed between expert CXR interpreters for whom more than 10 paired interpretations were available varied widely ranging from very poor to moderate (*κ* from −0.036 to 0.619). When restricted to the same subsets of scans as used by each pair of CXR interpreters, the kappa estimates for the two experts LUS interpreters were substantially higher (all >0.80 and most >0.90) than the corresponding kappa estimates for the expert CXR interpreters.

**Table 3a:** Interrater reliability among lung ultrasound (LUS) interpreters

LUS interpreter 1	LUS interpreter 2	*N*	*κ* estimate
Expert LUS 1	Expert LUS 2	220	0.914
Expert LUS final^a^	Onsite LUS Mozambique A	97	0.597
Expert LUS final^a^	Onsite LUS Mozambique B	97	0.206
Expert LUS final^a^	Onsite LUS Pakistan C	123	0.634
Expert LUS final^a^	Onsite LUS Pakistan D	123	0.619
Onsite LUS Mozambique A	Onsite LUS Mozambique B	97	0.196
Onsite LUS Pakistan C	Onsite LUS Pakistan D	123	0.983

^a^ Expert LUS final interpretations are identical to expert LUS 1 and expert LUS 2 interpretations when they agree, and when they did not agree, are determined by the majority interpretation involving a third tiebreaker expert LUS interpreter.

**Table 3b:** Interrater reliability among chest radiograph (CXR) expert interpreters

CXR interpreter 1	CXR interpreter 2	*N*	CXR *κ* estimate	LUS *κ* estimate^a^
Expert CXR 1	Expert CXR 2	118	0.401	0.958
Expert CXR 1	Expert CXR 3	10	1^b^	1
Expert CXR 1	Expert CXR 4	130	0.378	0.94
Expert CXR 1	Expert CXR 5	65	0.242	0.938
Expert CXR 1	Expert CXR 6	32	0.619	0.929
Expert CXR 2	Expert CXR 3	21	0.488	0.897
Expert CXR 2	Expert CXR 4	162	0.507	0.906
Expert CXR 2	Expert CXR 5	52	−0.036	0.876
Expert CXR 2	Expert CXR 6	62	0.403	0.934
Expert CXR 3	Expert CXR 4	24	0.318	0.909
Expert CXR 3	Expert CXR 5	4	1^b^	1
Expert CXR 4	Expert CXR 5	64	0.031	0.83
Expert CXR 4	Expert CXR 6	63	0.323	0.935

^a^ LUS kappa estimates are based on expert LUS 1 and expert LUS 2 evaluations of LUS from Table 3a, but restricted to the same subset of children as the expert CXR interpretations noted in the first two columns.

^b^ Kappa estimates of 1 for expert CXR interpreters 1 versus 3 and 3 versus 5 were based on small numbers of interpretations (10 and 4, respectively).

## 4 DISCUSSION

LUS demonstrated excellent interrater reliability between the expert LUS interpreters in diagnosing pneumonia. There was almost uniformly higher interrater reliability in diagnosing pneumonia between expert LUS interpreters than among onsite LUS interpreters with limited LUS training or among expert CXR interpreters. While Pakistan onsite LUS interpreters demonstrated high interrater reliability with each other and moderate interrater reliability with the expert LUS interpreters, Mozambique onsite LUS interpreters did not. Compared with the expert LUS interpreters, it appeared the Pakistan onsite LUS interpreters diagnosed pneumonia more frequently and the Mozambique onsite LUS interpreters diagnosed pneumonia less frequently. This discrepancy may be the result of increased disease burden and pathology in Pakistan or that more children were screened in the high‐volume urban hospital in Pakistan which resulted in the onsite LUS interpreters seeing more pathology on LUS compared with the onsite LUS interpreters in the low‐volume rural district hospital in Mozambique. In Mozambique, it may be that the onsite interpreters saw less pneumonia and less pathology on LUS, and, thus, were less familiar and less able to identify pneumonia on LUS, while in Pakistan, given their increased familiarity with abnormal LUS findings, the onsite interpreters overdiagnosed pneumonia on LUS compared to expert LUS interpreters.

In considering the differences in LUS performance between the sites in Mozambique and Pakistan and the potential use case for LUS as a diagnostic or screening tool in LRS, we need to consider factors, such as differing epidemiologies, severities, and presentations of disease, various comorbidities, such as HIV, malaria, and malnutrition, variable LUS operator/interpreter skill levels (nonphysician clinicians in Mozambique and technicians with previous ultrasound experience in Pakistan), and varying healthcare levels (low‐volume rural district hospital in Mozambique and high‐volume urban hospital in Pakistan), among others. For example, with minimal training, LUS may be an appropriate tool for use by technicians, while more training may be required for use by some clinicians,^15^, ^16^ particularly if they use this tool infrequently. Of note, all the onsite LUS operators after a short, limited but focused training were capable of obtaining quality LUS videos that the expert LUS interpreters could reliably interpret remotely. Thus, LUS operation and use may be feasible at many healthcare levels, but LUS interpretation may be more restricted in the absence of access to adequately trained interpreters or automated interpretation through machine learning. For point‐of‐care LUS to be successfully implemented for the diagnosis and management of pneumonia in LRS, the clinical environment and the appropriate amount of user training will need to be understood and addressed.

**Figure 4:**
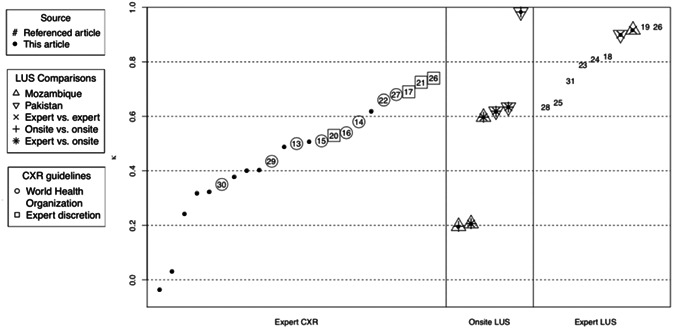
Kappa from this study and previous studies estimating interrater reliability of lung ultrasound (LUS) or chest radiograph (CXR). CXR kappa values from this study are restricted to those estimated using at least 20 children

Higher interoperator and interrater reliability for LUS interpretation than for CXR interpretation in identifying pediatric pneumonia is supported by the literature (Figure 4).^14^, ^16^, ^17^, ^18^, ^19^, ^20^, ^21^, ^22^, ^23^, ^24^, ^25^, ^26^, ^27^, ^28^, ^29^, ^30^, ^31^, ^32^, ^33^ We contrasted kappas observed in this study with kappas observed in the literature among other LUS and CXR interpreters. Kappas between LUS interpreters were 0.900 (in Pakistan) and 0.917 (in Mozambique) in this study (expert LUS interpreters) and ranged from 0.635 to 0.930 in the literature, whereas kappa between CXR interpreters ranged from −0.04 to 0.62 in this study and from 0.35 to 0.74 in the literature.

As demonstrated in this pilot with poor‐to‐moderate interrater reliability even among trained expert CXR interpreters, CXR itself is an imperfect reference standard, and, therefore, limited our ability to accurately assess LUS performance. Compelling evidence indicates that LUS may have greater sensitivity or specificity when compared with CXR, a diagnostic not readily available in LRS.^6^, ^22^, ^26^, ^28^, ^34^, ^35^ Initially, we considered analyzing the data using CXR as the reference standard (Table A5). However, CXR is a poor reference standard, and diagnosing pediatric pneumonia when there is no proven accurate and reliable gold standard is problematic.^8^ The true positive rate, false‐positive rate, positive predictive value, and negative predictive value for LUS in comparison to CXR in our study was a mix of relatively good as well as relatively poor statistics which we believe could be due to CXR being a relatively poor reference standard. Of note, despite being used widely for epidemiologic and vaccine effectiveness studies, the current WHO CXR interpretation methodology is not intended for clinical use; rather it is intended to serve as a research endpoint.^11^

Limitations to this pilot included the small sample size and sampling strategy, and employing different cadres of LUS users between the sites. This study design and analysis only included children who met the WHO IMCI chest‐indrawing pneumonia criteria, and, thus, did not allow us to investigate the sensitivity or specificity of these criteria themselves. Along with the different underlying pneumonia epidemiologies, because the study sites and the populations were different between the two and the sample sizes of the enrolled children were relatively small at each study site, there were limitations in the comparisons made between sites. Notably, of those screened, 81.8% in Pakistan versus 27.3% in Mozambique were not enrolled due to a lack of chest indrawing. This possibly could be explained by differences in healthcare‐seeking behavior at the two study sites and/or differences in screening procedures. Importantly, however, great care was undertaken at both sites to ensure that all eligibility criteria were met for enrollment. Finally, although all nonexperts, because the onsite LUS operators/interpreters were of different cadres at the two sites and had different backgrounds and levels of training before the study, this may have impacted their concordance with each other and with the expert LUS interpreters.

Among children with WHO IMCI chest‐indrawing pneumonia, expert interpreters may achieve substantially higher interrater reliability for LUS compared to CXR, and LUS could be the preferred reference standard, not only based on this study's findings, but also other studies. Identification of pneumonia that combines LUS imaging with clinical symptoms and signs could improve accurate diagnosis; however, there is still a need for adequately powered studies to validate the use of LUS for pediatric pneumonia diagnosis and a need for a gold standard. LUS operator/interpreter and site‐level variations are clearly factors in LUS performance, and more research is needed to better understand how LUS will perform in different LRS and how much training is necessary to achieve good to excellent interrater reliability.

## CONFLICT OF INTERESTS

The authors declare that there are no conflict of interests.

## AUTHOR CONTRIBUTIONS

Amy Sarah Ginsburg conceptualized the study, obtained research funding, designed the study and data collection instruments, coordinated and supervised data collection from the sites, interpreted the data, and drafted the manuscript. Jennifer L. Lenahan designed the study and data collection instruments, and coordinated and supervised data collection from the sites. Alessandro LaMorte and Giovanni Volpicelli provided input on the design of the study and designed the lung ultrasound methodology. Fyezah Jehan and Quique Bassat provided input on the design of the study and supervised teams that acquired the data. Among the authors, Rubao Bila, Lola Madrid, M. Imran Nisar, Pio Vitorino, Neel Kanth, Reyes Balcells, Benazir Baloch, Marta Valente, Rosauro Varo, and Naila Nadeem either oversaw or conducted the clinical procedures and acquisition of data. Jun Hwang and Susanne May performed the statistical analyses and interpreted the data, and drafted sections of the manuscript. All authors worked collaboratively to review and revise the manuscript and agree to be accountable for the work.

## Appendix

**Table A1:** Cross‐classified counts of expert lung ultrasound (LUS) and expert chest radiograph (CXR) pneumonia interpretations

		Mozambique				Pakistan			
		Expert CXR final^a^			*κ*	Expert CXR final^a^			*κ*
		Positive	Negative	Total		Positive	Negative	Total	
Expert LUS final^b^	Positive	9	6	15	0.453	11	45	56	0.178
	Negative	9	73	82		2	65	67	
	Total	18	79	97		13	110	123	

^a^ Expert CXR final interpretations are based on the majority interpretation for each subject among three expert CXR interpreters.

^b^ Expert LUS final interpretations are identical to expert LUS 1 and expert LUS 2 interpretations when they agree, and when they do not agree, are determined by the majority interpretation involving a third tiebreaker expert LUS interpreter.

**Table A2:** Cross‐classified counts of expert lung ultrasound (LUS) pneumonia interpretations

		Mozambique				Pakistan			
		Expert LUS 2			*κ*	Expert LUS 2			*κ*
		Positive	Negative	Total		Positive	Negative	Total	
Expert LUS 1	Positive	13	2	15	0.917	49	6	55	0.900
	Negative	0	82	82		0	68	68	
	Total	13	84	97		49	74	123	

**Table A3:** Cross‐classified counts of lung ultrasound (LUS) pneumonia interpretations comparing onsite to expert interpreters

		Mozambique							
		Onsite LUS A			*κ*	Onsite LUS B			*κ*
		Positive	Negative	Total		Positive	Negative	Total	
Expert LUS final^a^	Positive	7	8	15	0.597	2	13	15	0.206
	Negative	0	82	82		0	82	82	
	Total	7	90	97		2	95	97	

		Pakistan							
		Onsite LUS C			*κ*	Onsite LUS D			*κ*
		Positive	Negative	Total		Positive	Negative	Total	
Expert LUS final^a^	Positive	55	1	56	0.634	55	1	56	0.619
	Negative	22	45	67		23	44	67	
	Total	77	46	123		78	45	123	

^a^ Expert LUS final interpretations are identical to expert LUS 1 and expert LUS 2 interpretations when they agree, and when they do not agree, are determined by the majority interpretation involving a third tiebreaker expert LUS interpreter.

**Table A4:** Cross‐classified counts of lung ultrasound (LUS) pneumonia interpretations comparing onsite LUS interpreters

		Mozambique			
		Onsite LUS B			*κ*
		Positive	Negative	Total	
Onsite LUS A	Positive	1	6	7	0.196
	Negative	1	89	90	
	Total	2	95	97	

		Pakistan			
		Onsite LUS D			*κ*
		Positive	Negative	Total	
Onsite LUS C	Positive	77	0	77	0.983
	Negative	1	45	46	
	Total	78	45	123	

**Table A5:** Expert lung ultrasound (LUS) and expert chest radiograph (CXR) pneumonia determinations among children meeting World Health Organization Integrated Management of Childhood Illness chest‐indrawing pneumonia criteria by country, with true positive rate (TPR), false‐positive rate (FPR), positive predictive value (PPV), and negative predictive value (NPV) using CXR as the reference standard, with 95% confidence intervals (CI)

		CXR pneumonia determination						
		Negative	Positive	Total	TPR (95% CI)	FPR (95% CI)	PPV (95% CI)	NPV (95% CI)
Mozambique: LUS pneumonia determination	Negative	73	9	82	0.500 (0.469, 0.531)	0.076 (0.071, 0.085)	0.600 (0.323, 0.837)	0.890 (0.802, 0.949)
	Positive	6	9	15
	Total	79	18	97
Pakistan: LUS pneumonia determination	Negative	65	2	67	0.846 (0.796, 0.877)	0.409 (0.403, 0.415)	0.196 (0.102, 0.324)	0.970 (0.896, 0.996)
	Positive	45	11	56				
	Total	110	13	123				
